# The underappreciated contribution of Oskar Fischer to the knowledge of Frontotemporal Lobar Degeneration

**DOI:** 10.1590/1980-5764-DN-2024-0226

**Published:** 2025-02-10

**Authors:** Eliasz Engelhardt, Lea Tenenholz Grinberg

**Affiliations:** 1 Universidade Federal do Rio de Janeiro, Instituto de Neurologia Deolindo Couto, Instituto de Psiquiatria, Rio de Janeiro RJ, Brazil.; 2 University of California, Department of Neurology and Pathology, San Francisco CA, USA.; 3 Universidade de São Paulo, Faculdade de Medicina, Departamento de Patologia, São Paulo SP, Brazil.

**Keywords:** Frontotemporal Lobar Degeneration, Oskar Fischer, Neurodegenerative Diseases, Pick’s Disease, Degeneração Lobar Frontotemporal, Oskar Fischer, Doenças Neurodegenerativas, Doença de Pick

## Abstract

Frontotemporal Lobar Degeneration (FTLD) encompasses progressive neurodegeneration primarily affecting the frontal and/or temporal lobes, presenting in varied pathological entities. These pathologies lead to diverse clinical syndromes collectively known as Frontotemporal Dementia (FTD), which includes a behavioral variant, and primary progressive aphasia, with its non-fluent and semantic variants, and related conditions with parkinsonism or motoneuron disease features. The historical development of FTD concept is rooted in the pioneering work of Arnold Pick, who first described clinical cases of focal brain atrophy. Oskar Fischer significantly advanced this field by detailing the histopathological features of these conditions, such as the ‘spongiform cortical wasting’, and identifying characteristic neuronal changes such as the ‘ballooned cells’. Fischer’s contributions, alongside those of contemporaries like Alois Alzheimer, laid the foundation for understanding FTLD histopathology. Here, Fischer’s overlooked work is spotlighted, advocating for a greater recognition, which he rightfully deserves for his contributions to the field of neurodegenerative diseases.

## RESUMO

A Degeneração Lobar Frontotemporal (DLFT) compreende a neurodegeneração progressiva que afeta principalmente os lobos frontal e/ou temporal, apresentando-se em diversas entidades patológicas. Essas patologias levam a uma variedade de síndromes clínicas conhecidas coletivamente como Demência Frontotemporal (DFT), incluindo a variante comportamental, e a afasia progressiva primária, com suas variantes não fluente e semântica, bem como condições relacionadas, com parkinsonismo ou doença motora neuronal. O conceito da DFT tem suas raízes históricas no trabalho pioneiro de Arnold Pick, que primeiro descreveu casos clínicos de atrofia cerebral focal. Oskar Fischer avançou significativamente neste campo ao detalhar as características histopatológicas dessas condições, como a “atrofia cortical espongiforme”, e identificando alterações neuronais características, como as “células abalonadas”. As contribuições de Fischer, somadas às de Alois Alzheimer, lançaram as bases para a compreensão da histopatologia da FTLD. Aqui, o trabalho negligenciado de Fischer é destacado, e defende-se um maior reconhecimento, que ele legitimamente merece por suas contribuições no campo das doenças neurodegenerativas.

## INTRODUCTION

The term ‘Frontotemporal Lobar Degeneration’ (FTLD) is an umbrella concept that refers to progressive neurodegeneration predominantly of the frontal and/or temporal lobes, also affecting selective subcortical nuclei or motoneurons, and grouping different neuropathological entities^1,2,3^. The FTLD underlies varied clinical syndromes, determined by the distribution of the underpinning pathology, which makes up the ‘Frontotemporal Dementia’ (FTD) construct. The FTD/FTLD group usually manifests as one of the three prototypic clinical Frontotemporal Dementia (FTD) syndromes — the ‘behavioral variant’ (bvFTD), and two aphasic subforms (‘primary progressive aphasia’ [PPA]), a ‘non-fluent variant’ (nfvPPA), and a ‘semantic variant’ (svPPA). A proportion of patients may also display parkinsonism, and others motoneuron disease^1,4^.

The FTD concept emerged from the study of Arnold Pick (1851–1924), a psychiatrist born in Moravia (present-day part of the Czech Republic), of German-Jewish parents^5^. He described eight clinical cases of progressive aphasia, behavioral disorders, and dementia, with post-mortem gross changes featuring often asymmetric uni- or bilateral ‘circumscribed atrophies’ of the frontal and/or temporal lobes, often in an asymmetric manner (1892–1906). His main focus was the study of language disorders due to focal (lobar) brain atrophy, and the relevance of the distribution of the atrophic lobes in relation to the presented symptoms, irrespective of the underlying histopathology, as no microscopic examination was provided^6^.

Pick’s study was soon followed by a series of papers over the next two to three decades, most offering only minor contributions. However, two authors stand out for their significant, novel histopathological findings — Alois Alzheimer, with the description of microscopic aspects of two cases^7^, and Oskar Fischer, with his descriptions of the clinical, macro- and microscopic aspects of five cases^8^. The authors that assigned an eponym to the condition, first ‘Pick’s atrophy’ (*Picksche Atrophie*) by Abraham Gans^9^, who next designated the condition as ‘Pick’s disease’ (*Ziekte van Pick*) (1925), reaffirmed by Kimuri Onari and Hugo Spatz, also as ‘Pick’s disease’ (*Picksche Krankheit*), deserve to be mentioned^9,10^.

Following the 1920s, interest in Pick’s disease, seen as a very rare condition, became dormant until the appearance of the contributions of the Lund and Manchester groups (1980s and 1990s). Such studies revealed that the condition was not so rare. Based on this renewed interest, the term Pick’s disease evolved to include the terms ‘frontal lobe dementia,’ ‘dementia of frontal lobe type,’ and ‘Frontotemporal Lobar Degeneration’ (FTLD)^11^, ‘Pick’s complex’^12^, and ‘Pick’s lobar atrophy complex’^1^.

### The forgotten contributions of Fischer

The brilliant psychiatrist, and neuropathologist Oskar Fischer (1876–1942), was born in Bohemia (presently part of the Czech Republic), into a German-speaking Jewish family^6^ (Box 1)^6,13,14^. Recognizing his knowledge on luetic disease of the nervous system, he was invited to present the ‘anatomic part’ of “The Lues-Paralysis Question” (*Die Lues-Paralyse-Frage*) conference, at the ‘Yearly Meeting of the German Association of Psychiatry’ (1909), published in the same year. There he proposed the term ‘spongiform cortical wasting’ (*spongiöse Rindenschwund*) for the histopathology of the presented cases. He affirmed that such changes were also seen in other brain diseases, stating: “This cortical wasting is also remarkable in that it occurs with completely the same histological characteristics in other brain diseases, apart from paralysis.” He proceeded, citing five not-luetic dementia cases with such histopathologic changes^15^. Two years later he expanded on this in his paper “The spongiform cortical wasting, a special destructive process of the cerebral cortex” (*Der spongiöse Rindenschwund, ein besonderer Destruktionsprozess der Hirnrinde*) (1911), using classical staining techniques to describe the histopathology, in 17 dementia cases (12 luetic, and five non-luetic), out of approximately 3 hundred, all showing cortical wasting, but lacking ‘plaques’ and ‘tangles’^8^.(Box 2)

**Box 1.Fischer’s biography — excerpts.^6,13,14^**

Oskar Fischer was born on April 12, 1876, in Slaný, Kladno District, central Bohemia (presently part of Czech Republic), into a German-speaking Jewish family. He completed the medical formation at the Universities of Prague and Strasburg, and obtained his MD degree in Prague (1900). After that, he worked at the Department of Pathological Anatomy for two years, then moving to the Department of Psychiatry, where he stayed until 1919. This Department, between 1886 to 1921, was directed by Arnold Pick.

Fischer was assigned as physician-in-chief to the Division of Neurology and Psychiatry of the second Garrison Hospital in Prague, during WWI (1914-1918), where he treated many soldiers who had developed mental problems.

After WWI he returned to his position at the University, staying there until the Germans invaded Czechoslovakia (1939), at which time he was deposed from his teaching position for his Jewish heritage.

He was arrested by the Gestapo (early 1941), deported to, and imprisoned in a small fortress in Terezin (*Theresienstadt*), which the Nazis had turned into a concentration camp. There, he was severely ‘beaten to death’ by the guards, perishing on February 28, 1942. Fischer was buried in Prague, Czech Republic. His name is inscribed on one of the walls of the Pinkas Synagogue. One more victim of the Shoah (שואָה).

**Box 2. Fischer’s microscopic description of the ‘spongiform cortical wasting’ and other findings — excerpts.^8^**

According to Fischer the ‘spongiform cortical wasting’ (*spongiöse Rindenschwund*) occurs in several stages. The initial stage is characterized by small, circumscribed loosening of the cortical tissue with very sharply defined ‘spots’ (flecks) (*Flecke*), where the nervous tissue is severely rarefied. There, the ganglion cells have a significant decrease of their number or have disappeared, giving to such areas the appearance of a ‘clear spot’ (*heller Fleck*). The remaining ganglion cells are shrunken, their cell body stains somewhat darker (Nissl’s method), and the plasma appearing as a…network…a structure similar to a ‘gitter cell’ (*Gitterzelle*). The nuclei of these ganglion cells are very dark, irregular in shape, somewhat angular, and without a nucleolus. Additionally, ‘satellite cells’ (*Trabantzellen*), and glial nuclei may also be present…The ‘basic tissue’ [interstitial tissue] [neuropil] is loosened in a peculiar way (*eigenartiger Weise gelockert*), showing a very loose structure (*sehr lockerer Struktur*), [there] occurring cavities (*Hohlräume*) of different sizes, laying close together, parted by narrow bridges. The smallest cavities are scattered and spherical, or else these vesicular spaces (holes) usually lie together in groups, compressing one against the other, creating a honeycomb structure…

Regarding the ganglion cells, beside the description above, were also described in each senile case (‘presbyophrenic’ and ‘senile dementia’) (Table 1).

**Table 1:** Fischer’s cases — characteristics.^8,16^

Patient [gender-age]	Symptoms [diagnosis]	Gross pathology	Microscopic pathology
7. Mrs. XX [f-70 years]	(anamnesis lacking) **language:** aphasic symptoms **dx:** ‘senile dementia’ + ‘aphasia’	brain extensive atrophy (R more than L) R frontal and temporal lobes severe atrophy narrowed gyri, and very wide and deepened sulci white matter greatly reduced atrophic part of frontal lobes sharply delimited (*scharf begrenzt*), the borders running almost vertically on the medial and lateral surfaces temporal lobe L tip shrunken R ventricle very enlarged, more in the shrunken part of the brain, L less dilated [Figures not provided]	‘spongiform cortical wasting’ (*spongiösen Rindenschwund*) ganglion cells completely missing in the destroyed cortex ganglion cells with damaged Nissl corpuscles, and swelling of the ganglion cells (*Aufblähung der Ganglienzellen*) in the macroscopically unchanged cortex abundant neuroglial proliferation of the cortex in the shrunken areas white mater atrophic under the shrunken cortex
8. Anna Jirinec [f-75 years]	[duration before admission: 3½ years] **behaviour:** [early] doing wrong things such as pulling out vegetables in the garden, repeatedly running away from home, not recognizing her daughter, [later] dull, frequently unclean, not dressing-undressing; progressive dementia **neurologic exam:** hypoacusia [?], reacts to call if loud and near (word deafness [?]) **memory:** not provided **language:** speech: fluent, paraphasia word repetition [perseverative], senseless, evolving to word reduction, then jargon naming: recognizes some of the objects presented, says which are similar, knows how to use comprehension: severely impaired reading: with errors (alexia) writing: spontaneous very impaired (agraphia), copy spared (mostly mechanical) [servile] **dx:** ‘senile dementia’ + ‘aphasia’	brain with severe diffuse atrophy weight L<R temporal lobe severely affected [anterior part] [L more than R] gyri very shrunken (¼ of the normal thickness) (inner meninges over the parietal and occipital lobes with localized bloody infiltration) [frontal lobes moderate atrophy L more than R] [according to Pick’s pathologist Dr. Rubesch] [Figure 10: severe atrophy of the left temporal lobe]	diffuse senile atrophy of the brain severe atrophy of the L temporal lobe with gyri severely shrunken (L more than R) ‘spongiform cortical wasting’ abundant glial formation in the gyri of the L temporal lobe
9. Josef Ponec [m-68 years]	[duration before admission: mental illness for about 6 years] **behaviour:** [early] mostly moody and sad, sleeps badly, [late] restless and violent, especially at night. **observation:** [for 6 months] calm, cheerful mood, sings songs quietly to himself, sleeps little at night, walks around **neurologic exam:** disorientation (person, time, and place) **memory:** severely forgetful, confabulation, [‘presbyophrenic symptom complex’] **language:** not provided **dx:** ‘presbyophrenic dementia’	brain severe atrophy weight L>R frontal lobes severe atrophy - 1^st^ and 2^nd^ gyri very narrow, including the medial surface at R side, other gyri also somewhat narrowed. frontal lobes atrophy sharply delimited (*scharf begrenzt*) at the anterior central gyri ventricles widened, more R [Figures 11–13: reduction of the entire R hemisphere severe atrophy of both frontal lobes, more R, with sharp border between atrophic and no-atrophic parts]	severe ‘spongiform cortical wasting’ in atrophic cortex [R more than L] frontal cortex R ganglion cells almost completely missing in the affected gyri ganglion cells still present in these parts are small in size, round in shape and usually without processes frontal cortex L process milder degree ganglion cells of other parts of the brain slightly changed in some places no inflammatory changes and normal blood vessels [Figures: Plate III – Fig. 8]
10. Marie Rataj [f-54 years]	[duration before admission: 1 year] **behaviour:** [anamnesis] [early] stopped working, became careless, didn’t care about anything, spoke very little, did all sorts of ‘stupid things’, became unclean and often ran away from home **observation:** [for 2 years] always sat in the same place, soiled herself, let herself be fed, always smiled stupidly to herself **neurologic exam:** not provided **memory:** not provided **language:** speech: answers questions, initially monosyllabic, later mutism, only reacted by increasing her smile **dx:** ‘presbyophrenic dementia’	brain diffuse atrophy weight reduced L=R both frontal poles show severe atrophy anterior part of 1^st^, 2^nd^ and 3^rd^ gyri very narrowed on lateral and medial surfaces, and also orbital gyri cortex reduced on average to > ½ of normal thickness atrophied parts extend backwards in a gradual transition (not sharply) medulla beneath atrophic gyri greatly reduced basal ganglia small ventricles very enlarged [Figures non provided]	frontal lobes with severe ‘spongiform cortical wasting’ in shrunken cortex upper layers mostly affected, and in some places all layers foci of destruction with ganglion cells in various stages of changes and degeneration, and in places fairly well-preserved ganglion cells degenerating ganglion cells are rounded, have a swollen nucleus, no processes, no Nissl granules and no neurofibrils, and take on a deep black colour in the Bielschowsky’s preparation
11. Wenzel Pichler [m-44 years]	[anamnesis] history of lues infection and optic atrophy [later] seizures followed by transitory aphasia, and then always by confusion **neurologic exam:** entirely oriented, gives correct anamnesis; amaurosis [tabetic], pupillary rigidity, lack of reflexes, no Romberg, no ataxia, no sensory loss **observation:** complains that hears voices; at times remains in bed entirely rigid, and laconic; few months later develops dementia, urges to go home, gives stereotyped answers; stands for hours, mute, aside his bed; later becomes incoherent **dx:** ‘dementia’	R right hemisphere atrophic mainly due to severe frontal lobe shrinkage, affecting the 3^rd^ gyrus, anterior part of the 2^nd^ and 1^st^ frontal gyri, lateral and adjacent part of the medial optic nerves atrophied [Figures 14: severe atrophy of the right frontal lobe]	‘spongiform cortical wasting’ stage of rich glia proliferation process affecting almost the entire cortex in some places only the superficial layers are clearly affected no sign of paralytic destructive process

The ‘spots’, as mentioned, presented shrinkage and loss of cells. Besides, other changes were also described, comprising “swelling of the ganglion cells” (*Aufblähung der Ganglienzellen*)” (case #7); “ganglion cells…are small in size, round in shape and usually without *processes*” (*Ganglienzellen sind verkleinert, von rundlicher Gestalt und meist ohne Fortsätze*) (case #9), and “degenerating ganglion cells are rounded, have a swollen nucleus, no processes, no Nissl granules, and no neurofibrils” (*untergehenden Ganglienzellen sind rundlich, haben einen geschwellten Kern, keine Fortsätze, keine Nisslgranula und keine Neurofibrillen*) (case #10).

Further stages reveal neuroglial proliferation, and the appearance of ‘protoplasmic glial cells’ (*protoplasmatische Gliazellen*) [astroglia]. Further, there are stages with massive presence of “large multipolar glial cells and the formation of an abundant glial fiber felt” (*grosser multipolarer Gliazellen und Bildung eines reichlichen Gliafaserfilzes*) [astrogliosis]. This network is increasingly dense in the immediate vicinity of the blood vessels, which are relatively increased, giving to the focus a very characteristic spongiform aspect.

Lastly, he found that “the subcortical white matter under the atrophic areas of the cortex is reduced, and very poor in myelin”.

### Fischer’s non-luetic cases: clinical aspects

Fischer’s five non-luetic cases (Table 1)^8,16,17^ comprised two with ‘senile dementia’ (#7 and #8), two with ‘presbyophrenic dementia’ (#9 and #10), and one with a not defined ‘dementia’ (#11), showing lobar atrophy, as described by Pick, and where he provided the clinical, macro- and microscopic characteristics. Those with frontal lobe atrophy (#9, #10 and #11) predominantly presented behavioral manifestations, while one case (#7) with frontal and temporal lobe atrophy, and another one (#8) with bilateral temporal atrophy, presented aphasic symptoms.^8^

### Fischer’s non-luetic cases: gross neuropathology

All five non-luetic cases showed region-specific atrophy. Three cases had more pronounced atrophy in the frontal lobes (#9, #10 and #11), one in the right frontal and temporal lobes with additional atrophy in the left temporal lobe tip (#7), and one with bilateral temporal lobe atrophy, more severe on the left (#8). In two cases (#7 and #9) Fischer noted that the atrophic frontal gyri were “very sharply delimited” (*ganz scharf begrenzt*) from the normal-appearing central gyri^8^.

### Fischer’s non-luetic cases: microscopic aspects

Fischer provided a detailed description of the histopathological findings, primarily characterized by what he termed “spongiform cortical wasting” (*spongiösen Rindenschwund*) (Box 2). This condition involved complex microscopic changes affecting the cortical ganglion cells, neuroglia, neuropil, blood vessels, and subcortical white matter, similar to which he initially observed in luetic cases. Furthermore, one non-luetic case (#7) also presented peculiar neuronal changes, described as ‘swelling of the ganglion cells’ (*Aufblähung der Ganglienzellen*), and two cases (#9 and #10) presented ‘rounded ganglion cells’ (*Ganglienzellen…von rundlicher Gestalt*)^8,15^.

### Fischer’s examination of Pick’s cases

Fischer had the opportunity to examine microscopically the material of three Arnold Pick’s cases (1906). He identified stage IV and V ‘plaques’ (*Drusen*), with few ‘cub-shaped neurites’ (*Keulen*) (#5), ‘spongiform cortical wasting’ (#6), and features of ‘lues – Lissauer’ (#7) (1910, 1911)^8,16^. These findings proved the variable histopathological basis of seemingly similar cases of lobar atrophy.

## COMMENTS

The not-luetic cases presented by Fisher likely represent early examples of FTLD cases, where three (#9, #10, and #11) had a predominant frontal atrophy with behavioral manifestations, and may be classified, in present-day nomenclature, as bvFTD^18^, one (case #7) with right frontal and temporal lobe atrophy and involvement of the left temporal tip, presented with aphasia, potentially representing PPA^19^, though further subclassification is not possible due to limited data, and another case (#8) with bilateral temporal atrophy, exhibiting clinical features consistent with svPPA^4,19^. Pick has already published one of the cases (#8) (his case #2) (1904), but without microscopic examination^8,17^ (Table 2)^4,18,20^.

**Table 2:** Fischer’s cases classification on basis of clinical phenotypes — past and present.

Cases	Fischer’s diagnosis	Present days tentative diagnosis	Authors (diagnostic basis)
7. Mrs. XX	‘senile dementia’ + ‘aphasia’	aphasia [PPA (?)]	Gorno-Tempini et al.^4^
8. Anna J.*	‘senile dementia’ + ‘behavioural symptoms’ [early]+ ‘aphasia’	bvFTD + svPPA [overlap (?)]	Rascovsky et al.^18^ Gorno-Tempini et al.^4^ Kertesz and Munoz^20^
9. Josef P.	‘presbyophrenic dementia’ + ‘behavioural symptoms’	bvFTD	Rascovsky et al.^18^
10. Marie R.	‘presbyophrenic dementia’ + ‘behavioural symptoms’	bvFTD	Rascovsky et al.^18^
11. Wenzel P.	[lues**] + ‘dementia’+ ‘behavioural symptoms’	bvFTD	Rascovsky et al.^18^

Notes: *Pick’s case 2^17^;

**history of lues infection — no sign of paralytic lesions.

Fischer was the first to describe a sharp delimitation of the atrophic frontal lobe gyri from the normal-appearing central gyri by describing the gross pathologic anatomy in two of his cases (#7 and #9) of lobar atrophy^8^. Later, Hugo Richter confirmed this finding, noting the “sharp delimitation of the atrophic process” (*scharfe Abgrenzung des atrophischen Prozesses*)^21^. This sharp cortical atrophy later became known as ‘knife-blade’^22^ or ‘knife-edge’ pattern^23^.

Microscopic examination of the brain tissue revealed a range of histopathological changes, such as the ‘spongiform cortical wasting’, described by Fischer for this group of diseases for the first time^8^. Later, Emil Altman described similar changes, which he called “spongy state” (*Status spongiosus*)^24^. The term “spongiosis” was subsequently introduced by the Lund and Manchester workgroups to describe changes primarily in the upper cortical layers (I-III), forming the histopathological basis of “frontal lobe degeneration” (FLD)^22^. Finally, the descriptive term ‘microvacuolation’ came to replace the former designations^1,3^.

Some authors attribute the first histopathologic study of a case compared to Pick’s disease to Erwin Stransky. He described mild neuronal changes involving all cortical layers, accompanied by perivascular glial condensation, which was more prominent in subpial regions than in deeper cortical areas. He also noted changes around the vessels entering the cortex from the pia mater, as well as superficial cortical depressions and elevations, and absence of plaques or signs of syphilis^25^. However, Stransky’s description appears consistent with Alzheimer’s ‘perivascular gliosis’ (*perivasculäre Gliose*) (1897)^26^, suggesting a pattern more characteristic of small vessel disease rather than neurodegeneration.

Additionally, Fischer was the first to describe what are now referred to as ‘swollen’ or ‘ballooned’ neurons, and ‘Pick’s cells,’ which are associated with certain conditions within the FTLD spectrum, particularly those involving tau inclusions^20,27,28^. In his work, Fischer identified distinctive neuronal changes, describing ‘swelling of the ganglion cells’ (*Aufblähung der Ganglienzellen*) in one case and ‘rounded ganglion cells’ (*Ganglienzellen…von rundlicher Gestalt*) in two others^8^. This observation was soon corroborated by Walther Spielmeyer (1912), who noted that “cells are often strongly bulged…and some exhibit…swelling of the cell body” *(Zellen sind oft ausgebaucht und in manchen…Aufblähung des Zelleibes*)^29^.

Alois Alzheimer (1864–1915) was the first to describe another neuropathological hallmark of FTLD-Pick’s disease, the so-called Pick’s bodies. He examined two cases with brain atrophy similar to those described by Pick and identified distinct neuronal changes marked by round intracytoplasmic inclusions, which he termed “argentophilic balls” (*argentophile Kugeln*), visible with Bielschowsky’s silver impregnation technique^7,20,28^. Additionally, when the nervous tissue was stained with alcohol-toluidine blue (Nissl’s method), the neurons exhibiting these fibrillary changes could be recognized by their “distinct overall shape” and the peculiar “dull-shining color at the site of the ‘argentophilic mass’^7^. This description suggests that these neurons stained positively with silver impregnation and negatively with toluidine blue, although subsequent authors, such as Onari and Spatz^10^, and later others, also credited Alzheimer with the description of ‘ballooned neurons’. However, no such description is found in his original paper on the subject^7^, apparently a misunderstanding of Alzheimer’s findings.

Thus, the initial histopathologic findings that became markers of the lobar atrophy condition must be credited to Fischer (the ‘wasting’ and the Pick’s cells) and Alzheimer (the Pick’s bodies), who provided the pivotal classic descriptions, each having published a paper on the subject in the same year and month (December, 1911)^7,8^.

While it is undeniable that Pick laid the foundation for what would later be recognized as FTD/FTLD, or Pick’s complex, Fischer’s contributions to understanding this group of diseases are equally significant. Fischer was a pioneer in recognizing the critical role of histopathological examination in diagnosing neurodegenerative conditions with accuracy.

He understood that macroscopic findings, such as lobar atrophy, could be present in various neuropathological entities, and that a deeper histological analysis was essential for differentiation. Using the histological techniques available in his time, Fischer was able to distinguish the group of diseases marked by ‘spongiform cortical wasting’, and the presence of ‘ballooned cells’^8^. His microscopic findings established a histopathological identity for these conditions that are now understood as key features of FTLD.

Fischer’s perception foreshadowed the significant histopathological variability that was later explored in greater detail with the advent of advanced histochemical and immunohistochemical techniques, expanding the knowledge of this complex group of diseases^30^.

Despite his pioneering work and major contributions to the field of neurodegenerative diseases, it remains puzzling why Fischer’s work has not received the recognition it deserves, even today. Fortunately, his legacy survives in his papers, which are gradually being rediscovered. It is hoped that this renewed attention will ensure that Fischer’s contributions to neuropathology are finally acknowledged.
